# Supplementary material for: The Prevalence of Anemia and Its Associated Factors among Older Persons: Findings from the National Health and Morbidity Survey (NHMS) 2015
Source: Int J Environ Res Public Health. 2022 Apr 20;19(9):4983. doi: 10.3390/ijerph19094983 (PMC9101117; doi:10.3390/ijerph19094983)
Supplement: Supplementary file 1 [file ijerph-19-04983-s001.zip › ijerph-1637568-supplementary.pdf]

Supplementary Table S1. Multivariable analysis of the factors associated with anemia among Malaysian older persons (≥60 years old); NHMS 2015

Parameters	Older person; n=3556								Older person having diabetes mellitus; n=1404								Older person not having diabetes mellitus; n= 2152							
	SLR				MLR				SLR				MLR				SLR				MLR			
	OR	(95% CI) LL	(95% CI) UL	p-value	aOR#	(95% CI) LL	(95% CI) UL	p-value	OR	(95% CI) LL	(95% CI) UL	p-value	aOR#	(95% CI) LL	(95% CI) UL	p-value	OR	(95% CI) LL	(95% CI) UL	p-value	aOR#	(95% CI) LL	(95% CI) UL	p-value
Age group																								
60-69	**1.00**	**-**	**-**		1.00	-	-		1.00	-	-		1.00	-	-		1.00	-	-		1.00	-	-	
70-79	**1.52**	**1.30**	**1.77**	**<0.001**	**1.42**	**1.21**	**1.66**	**<0.001**	**1.56**	**1.24**	**1.98**	**<0.001**	**1.38**	**1.08**	**1.76**	**0.009**	**1.49**	**1.22**	**1.81**	**<0.001**	**1.46**	**1.19**	**1.80**	**<0.001**
80 and above	**3.10**	**2.40**	**4.00**	**<0.001**	**2.64**	**2.00**	**3.47**	**<0.001**	**3.17**	**2.03**	**4.93**	**<0.001**	**2.48**	**1.56**	**3.94**	**<0.001**	**3.17**	**2.31**	**4.34**	**<0.001**	**2.89**	**2.05**	**4.07**	**<0.001**
Sex																								
Male	1.00	-	-		-	-	-	-	1.00	-	-		-	-	-	-	1.00	-	-		-	-	-	-
Female	1.15	1.00	1.31	0.051	-	-	-	-	1.15	0.93	1.43	0.202	-	-	-	-	1.12	0.94	1.34	0.197	-	-	-	-
Ethnicity																								
Malays	1.00	-	-		-	-	-	-	1.00	-	-		-	-	-	-	1.00	-	-		-	-	-	-
Non-Malays	0.99	0.86	1.14	0.872	-	-	-	-	1.18	0.94	1.47	0.155	-	-	-	-	0.89	0.74	1.07	0.221	0.85	0.70	1.03	0.100
Marital Status																								
Currently not married	**1.35**	**1.17**	**1.56**	**<0.001**	-	-	-	-	**1.48**	**1.18**	**1.86**		1.23	0.97	1.55	-	**1.27**	**1.05**	**1.53**	**0.015**	-	-	-	-
Currently married	**1.00**	**-**	**-**		-	-	-	-	**1.00**	**-**	**-**	**0.001**	-	-	-	0.092	**1.00**	**-**	**-**		-	-	-	-
Location																								
Urban	1.00	-	-		-	-	-	-	1.00	-	-		-	-	-	-	1.00	-	-		-	-	-	-
Rural	1.03	0.90	1.18	0.639	-	-	-	-	1.00	0.81	1.24	0.971	-	-	-	-	1.07	0.90	1.28	0.429	-	-	-	-
Education Level																								
Primary and below	**1.00**	**-**	**-**		-	-	-	-	**1.28**	**1.01**	**1.62**		-	-	-	-	**1.41**	**1.14**	**1.74**		-	-	-	-
Secondary/higher	**1.33**	**1.14**	**1.56**	**<0.001**					**1.00**	**-**	**-**	**0.043**					**1.00**	**-**	**-**	**0.002**				
Diabetes																								
No	**1.00**	**-**	**-**		**1.00**	-	-		-	-	-	-	-	-	-	-	-	-	-	-	-	-	-	-
Yes	**1.29**	**1.13**	**1.49**	**<0.001**	**1.30**	**1.13**	**1.51**	**<0.001**	-	-	-	-	-	-	-	-	-	-	-	-	-	-	-	-
Hypertension																								
No	1.00	-	-		-	-	-	-	1.00	-	-		-	-	-	-	1.00	-	-		-	-	-	-
Yes	1.07	0.92	1.25	0.357	-	-	-	-	1.18	0.89	1.55	0.246	-	-	-	-	0.96	0.80	1.15	0.646	-	-	-	-
Hypercholesterolemia																								
No	**1.00**	**-**	**-**		1.00	-	-		1.00	-	-		1.00	-	-		1.00	-	-		-	-	-	-
Yes	**0.86**	**0.74**	**0.99**	**0.037**	0.87	0.75	1.01	0.075	0.81	0.64	1.03	0.080	-	-	-	-	0.86	0.71	1.04	0.114	-	-	-	-
Difficulty in Seeing																								
No	**1.00**	**-**	**-**		-	-	-	-	**1.00**	**-**	**-**		**1.00**	**-**	**-**	**-**	1.00	-	-		-	-	-	-
Yes	**1.28**	**1.11**	**1.47**	**0.001**	-	-	-	-	**1.60**	**1.29**	**1.99**	**<0.001**	**1.29**	**1.02**	**1.63**	**0.037**	1.07	0.89	1.29	0.471	-	-	-	-
Difficulty in Hearing																								
No	**1.00**	**-**	**-**		1.00	-	-		**1.00**	**-**	**-**		-	-	-	-	1.00	-	-		1.00	-	-	
Yes	**1.29**	**1.10**	**1.52**	**0.002**	0.84	0.70	1.01	0.071	**1.51**	**1.18**	**1.93**	**0.001**	-	-	-	-	1.13	0.91	1.41	0.225	0.80	0.61	1.04	0.092
Difficulty in Walking																								
No	**1.00**	**-**	**-**		1.00	-	-		**1.00**	**-**	**-**		1.00	-	-		**1.00**	**-**	**-**		1.00	-	-	
Yes	**1.65**	**1.43**	**1.89**	**<0.001**	**1.31**	**1.11**	**1.54**	**0.001**	**1.91**	**1.54**	**2.37**	**<0.001**	**1.50**	**1.18**	**1.91**	**0.001**	**1.44**	**1.20**	**1.73**	**<0.001**	1.23	0.98	1.54	0.071
Difficulty in Self-care																								
No	**1.00**	**-**	**-**		1.00	-	-		**1.00**	**-**	**-**		-	-	-	-	**1.00**	**-**	**-**		**1.00**	**-**	**-**	
Yes	**2.30**	**1.84**	**2.87**	**<0.001**	**1.58**	**1.22**	**2.05**	**<0.001**	**2.30**	**1.65**	**3.21**	**<0.001**	-	-	-	-	**2.23**	**1.65**	**3.02**	**<0.001**	**1.87**	**1.30**	**2.69**	**0.001**
Difficulty in																								

Remembering																								
No	**1.00**	**-**	**-**		-	-	-	-	**1.00**	**-**	**-**		-	-	-	-	1.00	-	-		-	-	-	-
Yes	**1.35**	**1.16**	**1.58**	**<0.001**	-	-	-	-	**1.70**	**1.33**	**2.16**	**<0.001**	-	-	-	-	1.14	0.92	1.40	0.228	0.78	0.60	1.01	0.064
Difficulty in Communicating																								
No	**1.00**	**-**	**-**		-	-	-	-	**1.00**	**-**	**-**		-	-	-	-	**1.00**	**-**	**-**		-	-	-	-
Yes	**1.63**	**1.32**	**2.01**	**<0.001**	-	-	-	-	**1.81**	**1.30**	**2.52**	**0.001**	-	-	-	-	**1.53**	**1.17**	**2.01**	**0.002**	-	-	-	-

SLR: Simple Logistic Regression
MLR: Multivariable Logistic Regression
aOR#: adjusted with all the variables
Hosmer Lemeshow goodness of fit test (p value: 0.588)
